# Annotations

**Published:** 1908-08-29

**Authors:** 


### ANNOTATIONS.

### Cancer and Tubercle.

Cancer and tubercle have been considered as in many respects antagonistic. Writers have often remarked on the great rarity of their clinical coincidence; the commonest sites of origin of cancer—namely, the stomach, liver, womb, and breast—are comparatively seldom the seats of primary, or, indeed, of secondary, tuberculosis; while, on the other hand, the lung, so often tuberculous, hardly ever originates any form of malignant disease. Young people are often attacked by consumption, seldom by cancer. In his recently published book, "The Natural History of Cancer," W. R. Williams, while admitting the above facts, seeks to establish some inter-relations of these two great diseases. In 136 consecutive necropsies of cancerous women, he found macroscopically obsolete pulmonary tubercle in 12.5 per cent., a proportion about three times as great as that recorded by Heitler in an unselected series. Apical pleural adhesions were also very common. He thinks that while active tuberculous disease of any significance is very rare in the cancerous, malignant disease not infrequently follows or coincides with the healing of tubercle. More conclusive, perhaps, are his investigations into the frequency of a phthisical family history in cancer patients, because these can be compared with the records of other workers. Williams's analysis of 134 cases of mammary cancer shows a family history of consumption in 55 per cent. This percentage is very greatly in excess of any found in the non-tuberculous generally, and almost reaches the highest figures—*e.g.* those of R. E. Thompson, noted with female consumptives. Long study, he says, of the family history of cancer patients has convinced him that most of them are the surviving members of tuberculous families. If these and other ideas of the author be correct, there is some ground for his rather provocative remark that "those pathologists whose horizon does not extend beyond cells and microbes have overlooked the chief factor in the cancer problem—that is to say, predisposition."

### Doctors and their Fees.

As is only natural at this time of the year, a recent incident in the St. Pancras Coroner's Court has received much publicity. The alleged refusal by a doctor to attend a man who had cut his throat until the fee had been guaranteed has brought to light the curious attitude of the public towards doctors and their fees. The well-known fact that medical men almost invariably obey an urgent summons, quite irrespective of possible payment for their services, has led many people to suppose that medical assistance can be demanded as a right in real or supposed emergencies. This custom of prompt disinterested attention on the part of the profession is not likely to be altered, however much it is abused; but it is just as well that the incident of last week has been brought prominently before the public, for it may perhaps lead to a better appreciation of the vast amount of gratuitous work that is daily performed by medical practitioners. The temperate comments of the coroner were no doubt only what the occasion demanded; but there are many ways of putting facts before jurymen, and the medical profession owes something to Mr. Schröder for his appreciation of the usual custom of doctors in cases of "life and death" summonses and for his warm tribute to the general humanity of the profession. Medical men as a whole are pleased to render that aid which they alone can give in times of grave physical danger, and the question of payment or otherwise is not the first and only thought that crosses their mind when an urgent message comes. But they are entitled to ask of the public that this altruistic practice of the profession should at least be recognised for what it is. During the past few days some of the newspapers have drawn attention, almost with the air of propounding a new truth, to the fact that medical men have rates and taxes to pay, homes to support, and appearances to keep up, just like other people. To many laymen, indeed, there seems something inhuman and revolting in the circumstance of a doctor haggling for payment; and therein lurks the shadow of a compliment. But implied compliments avail little when times are hard.

### Maxims of an Old Physician.

Under the signature "Dr. R. L.," a recent number of *Le Scalpel* contains a collection of maxims for young practitioners as enunciated by an old physician. The following is a somewhat free translation:—(1) Look after a patient as you would be looked after yourself. (2) A good doctor is he who is able to treat each case on its own merits. (3) Never show anger towards a child. (4) Show your patient that you understand the cause of his suffering, but do not make him as wise on the point as yourself. (5) If your patient sends for you, go; but do not be eager to anticipate his desire for your presence. (6) Look after details; sometimes the effects of a massage will be judged by the amount of talc you have powdered on to the part. (7) A doctor in a carriage never looks a bungler. (8) When called to a patient, let him be under the impression that you have already dealt successfully with a similar case. (9) Every man is in need of a little of the mysterious, more especially when he is sick. (10) Be in the habit of making people understand that you have, because of your profession, a different conception of things from theirs. (11) If payment is offered on the spot, accept it always. This procedure is the usual one in England, and the English pass for a practical people. (12) When sending in your bill, remember that patients will weigh your knowledge against the number of five-franc pieces asked for. (13) A doctor is never allowed to be in a hurry, except during an operation and when his patient is asleep. (14) Explain nothing to a patient who has some smattering of things medical. (15) He who demands small fees passes for an ignoramus; he who demands too much is a money-grabber. (16) Beware of women and their tittle-tattle. (17) If you keep a cool head as regards your patient, he will keep a cool one as regards yourself.

